# Rhythm Perception in Speakers of Arabic, German and Hebrew

**DOI:** 10.1007/s10936-024-10121-5

**Published:** 2025-01-05

**Authors:** Osnat Segal, Tom Fritzsche, Anjali Bhatara, Barbara Höhle

**Affiliations:** 1 Department of Communication Disorders, The Stanley Steyer School of Health Professions, Faculty of Medical and Health Sciences, Tel-Aviv University, Tel Aviv, Israel; 2 Sagol School of Neuroscience, Tel-Aviv University, Tel Aviv, Israel; 3 Department of Linguistics, University of Potsdam, Potsdam, Germany; 4 Integrative Neuroscience and Cognition Center, CNRS - Université Paris Cité, Paris, France

**Keywords:** Iambic-Trochaic Law, Lexical stress, Phrasal stress, Palestinian–Arabic, Hebrew, German

## Abstract

Rhythm perception in speech and non-speech acoustic stimuli has been shown to be affected by general acoustic biases as well as by phonological properties of the native language of the listener. The present paper extends the cross-linguistic approach in this field by testing the application of the iambic-trochaic law as an assumed general acoustic bias on rhythmic grouping of non-speech stimuli by speakers of three languages: Arabic, Hebrew and German. These languages were chosen due to relevant differences in their phonological properties on the lexical level alongside similarities on the phrasal level. The results show Iambic-Trochaic-Law (ITL) conforming weak–strong grouping for duration-cued acoustic salience. However, only German participants judged intensity-varying sequences as strong–weak; no grouping preferences were found for speakers of Arabic and Hebrew. Overall these results suggest that prosodic properties of the phonological phrase and of the lexical level of the native language show differing effects on rhythmical grouping.

**Supplementary Information:**

The online version contains supplementary material available at 10.1007/s10936-024-10121-5.

## Introduction

Human auditory perception is affected by the sound properties of the listener’s native language. One well-known example of this is the difficulty that adult second language learners may have in distinguishing sounds in their second language that fall into a single phonological category of their first language (Escudero & Williams, 2011; Tyler et al., 2014). These difficulties are the result of the perceptual system’s attunement to the sound properties of the native language which leads to an increase in sensitivity to those sound properties that are relevant in the native language’s phonological system and a decrease in sensitivity to irrelevant sound properties. This process goes hand in hand with infants’ earliest language development and is an indication that speech perception is modulated by the phonological system of the native language. Strikingly, its consequences on perception are hard to reverse at an older age (e.g., Höhle et al., 2009).

Perceptual attunement affects not only segmental sound properties but also suprasegmental properties such as the perception of lexical stress (e.g., Dupoux et al., 1997, 2001; Segal & Kishon-Rabin, 2019), of lexical tone (e.g., Götz et al., 2018; Mattock & Burnham, 2006), and speech rhythm (e.g., Bhatara et al., 2013). For the latter area, recent discussions centre around the question of how potential general acoustic biases interact with the emergence of perceptual mechanisms that are adjusted to the sound properties of the native language. In this respect, a well-known acoustic bias is the so-called Iambic-Trochaic-Law (ITL; Bolton, 1894; Hayes, 1985, 1995; Woodrow, 1909). This law states that sound sequences varying in intensity are perceptually grouped to strong–weak patterns with the more salient (i.e., louder) element at the beginning of the pair while sequences varying in duration are grouped to weak–strong patterns with the more salient (i.e., longer) element at the end of the pair. Many studies have provided evidence that listeners apply the ITL when listening to speech as well as non-speech stimuli (e.g., Bhatara et al., 2013; Bolton, 1894; Hay & Diehl, 2007; Rice, 1992; Woodrow, 1909), and that the acoustic perception of some non-human species is also guided by this bias (de la Mora et al., 2013; Spierings et al., 2017). Pitch as another acoustic cue was shown to have effects comparable to intensity, leading to a strong–weak (high-low) grouping (Bion et al., 2011; Langus et al., 2016).

The current study contributes to this research landscape by adding evidence from two languages that have not yet been tested concerning the effect of the ITL on listeners’ grouping preferences: Arabic and Hebrew. Rhythmic grouping of non-speech stimuli by speakers of these languages was compared to that of speakers of German who had shown ITL-conforming grouping patterns for these stimuli in a previous study (Bhatara et al., 2016).

### Cross-Linguistic Findings on Perceptual Effects of the ITL

One of the first studies that compared ITL effects cross-linguistically was conducted by Hay and Diehl (2007). They tested English- and French-speaking adults with syllable and tone sequences varying in intensity or duration and found ITL-conforming performance without significant differences between the two language groups or between the stimulus types. In contrast, Iversen et al. (2008) report differences between native speakers of English and Japanese in the grouping of non-speech tone sequences varying in intensity or duration. While English-speaking participants showed ITL-conforming grouping, Japanese-speaking participants grouped the sequences as strong–weak for both intensity and duration changes, indicating non-ITL-conforming response in the duration condition. Iversen et al. (2008) consider syntactic differences on the phrase level between these two languages as relevant here: while English has a functor–content word order, the functor follows the content word in Japanese. As functors are typically shorter than content words this leads to a dominance of short-long patterns in English and the reverse in Japanese.

Bhatara et al. (2013) compared speakers of German or French on their rhythmic grouping of speech sequences varying in pitch, intensity, duration, or not varying at all in one of these parameters as a control condition. The German speakers consistently grouped the intensity- or pitch-varying sequences as strong–weak and the duration-varying sequences as weak–strong. In addition, they also judged the control sequences (without acoustic cues for prominence) as strong–weak. The French participants showed consistent ITL-conforming grouping only for the sequences that varied in intensity, while grouping in the pitch and duration condition was less consistent and no strong–weak bias occurred in the control condition. The fact that these results are not consistent with those from Hay and Diehl (2007) may be due to methodological differences: while Hay and Diehl presented only one single token of a syllable (ga) with pauses of 200 ms between the single instances, the speech stream in Bhatara et al. consisted of segmentally varying syllables without any pauses. Bhatara et al. (2013) consider the presence or absence of lexical stress as relevant for the cross-linguistic difference they found between the speakers of German and French: German has contrastive lexical stress with some words differing only by the position of the stressed syllable while French has no lexical stress but phrase-final stress, with duration as the most relevant acoustic cue.

Bhatara et al. (2016) also compared rhythmic grouping by native speakers of German and French but used non-speech stimuli that they called “instrumental syllables”. These stimuli were created by combining the spectral information of one set of musical instruments with the temporal information of another set of musical instruments. Following the discrepancy between the previous findings this study also investigated the effects of stimulus variability by either using only one or 16 different instrumental syllables. German speakers showed no effect of variability with ITL-conforming responses in both conditions. However, the French participants responded according to the ITL only in the low variability condition while their responses were not different from chance in the high variability condition, independent from the acoustic cue. Interestingly, the German participants again showed a strong–weak bias in a control condition without any intensity or duration cues.

Crowhurst (2016) compared grouping of speech sequences either varying in intensity or duration by speakers of English and Spanish – both languages with trochaic lexical stress and phrase final stress. She found no cross-linguistic differences in the grouping of intensity-varied sequences as both language groups showed strong–weak groupings in that condition. However, in the duration condition English speakers showed stronger ITL-conforming weak–strong groupings than the speakers of Spanish. Crowhurst suggests that this reflects cross-linguistic differences since English has more words with final stress (typically encoded by duration) and stronger phrase final lengthening than Spanish.

Stronger evidence for cross-linguistic variation concerning the ITL was found in a study by Crowhurst and Teodocio Olivares (2014) that tested speakers of Zapotec (a language spoken in Mexico) on their grouping of intensity or duration varied stimuli. Zapotec was chosen as a language of interest since it has lexical stress with a dominance of trochaic patterns but duration as the most relevant acoustic correlate. Results showed a bias to strong–weak groupings in the Zapotec speakers which was more pronounced in the intensity condition but not overruled by duration. In contrast, speakers of English tested with the same materials and procedure displayed ITL-conforming groupings for both cues. The authors propose that the lexical stress system of Zapotec with duration as the main cue for stress which typically falls on the first syllable of a root is relevant for the finding that duration-varied sequences were not grouped according to the ITL by the Zapotec speakers.

Langus et al. (2016) extended the cross-linguistic perspective by comparing the performance of Italian, Persian and Turkish speakers with stimuli varying in either pitch or duration. These languages were selected because of their systematic differences on the level of the phonological phrase and in lexical stress. Lexical stress is determined by the prosodic foot structure of a language with prominence either on the right or left edge of the foot typically signalled by a combination of intensity and duration. Phrasal stress is connected to syntactic properties of word order (head–complement order that specifies for instance the relative order of object and verb in a language). Across languages, the syntactic complement carries prosodic prominence if the syntactic phrase corresponds to a phonological phrase, but the acoustic cues for prominence vary with the position of the complement: if it is phrase initial, prominence is cued by intensity and pitch, if it is phrase final prominence is cued by duration (Nespor et al., 2008). Italian has phrase final stress with duration as the main acoustic cue for salience while Persian and Turkish have phrase initial stress cued by pitch and intensity. On the lexical level, most Italian words carry stress on the penultimate syllable cued by duration. In Persian, the last stressable syllable in the word carries stress signalled by pitch and intensity. Turkish uses the same acoustic cues as Persian does but has word final stress. The results by Langus et al. (2016) showed no cross-linguistic differences in the responses to speech stimuli varying in pitch: speakers of all three languages grouped them as strong–weak. However, cross-linguistic differences occurred for the stimuli varying in duration. Only the Italian speakers grouped them ITL-conforming as weak–strong while Persian and Turkish speakers showed a preference for a strong–weak grouping, thus not responding as predicted by the ITL. Hence, the results of this study suggest cross-linguistic differences only for duration while the pitch cue leads to trochaic grouping independent of the language background. In contrast, the results in the duration condition mirror the properties across the three languages on the level of the phonological phrase with Persian and Turkish showing phrase-initial prominence cued by pitch and intensity, Italian showing phrase-final prominence cued by duration. Since duration is not a cue for stress in Persian and Turkish, the authors assume that the dominant strong–weak pattern was perceived even when duration was the cue that made one syllable more salient. However, these cross-linguistic differences only occurred with speech stimuli. In a second experiment that used sine-wave speech, in which the phonetic information necessary to identify the segments was removed, ITL-conforming groupings were shown for pitch and duration by all three language groups.

Another area to investigate language-specific effects on the ITL are bilingual speakers. Molnar and colleagues (2016) tested Spanish monolingual and Spanish–Basque bilingual speakers with tone sequences. All language groups showed strong–weak groupings for intensity-varied sequences but the grouping of duration-varied sequences was modulated by the language status. While the Spanish monolinguals showed ITL-conforming weak–strong groupings the bilinguals showed more strong–weak groupings which increased with their dominance in Basque. These differences are in line with differences on the phrasal level between the two languages. Spanish has a head–complement order with phrase final prominence while Basque has a complement–head order with phrase initial prominence. On the other hand, Boll-Avetisyan et al. (2020) found that simultaneous bilingual German–French speakers showed the same patterns of ITL-conforming grouping for speech sequences as monolingual German which was stronger than that of monolingual French speakers. Interestingly, language exposure during childhood did not modulate the bilinguals’ performance.

Wagner and colleagues (Moghiseh et al., 2023; Wagner, 2022) present a different view on the ITL which, according to their proposal, only emerges when duration and intensity are dissociated and have extreme values as it is typical for the stimuli used in experiments testing the ITL. However, this dissociation is not characteristic for natural speech where intensity and duration are typically correlated in cueing prominence while they encode grouping when they are not correlated. Hence, the listener must parse the speech stream along both acoustic dimensions and consider their interactions to exploit the cues for rhythmic grouping which is reflected in findings of experiments that used sequences in which intensity and duration were modulated simultaneously (c.f. also Crowhurst, 2016; Crowhurst & Teodocio Olivares, 2014).

### The Current Study

The previous research suggests that prosodic properties at the lexical as well as at the phrasal level may modulate effects of the ITL on grouping. Some studies show cross-linguistic differences between languages that mainly differ on the lexical level (Bhatara et al., 2013; Crowhurst & Teodocio Olivares, 2014) while others attribute cross-linguistic differences to the phrase level prosody (Iversen et al., 2008; Langus et al., 2016; Molnar et al., 2016). Furthermore, non ITL-conforming results only appeared for the duration but not for the intensity cue. This may suggest that the effects of the two cues have different sources – one being a universal principle and the other emerging via language experience which is in line with findings of a developmental advantage for intensity or pitch compared to duration in infants (Bion et al., 2011; Hay & Saffran, 2012). So far, research on perceptual effects of the ITL has mainly focused on European languages thus, one of the main goals of the current study is to broaden the view on further languages beyond the European focus. Therefore, speakers of Arabic and Hebrew—representing two languages from the Semitic group that was not previously investigated within the ITL research—were tested and compared to speakers of German—a language for which ITL-conforming grouping has repeatedly been shown in previous research. Investigating these languages was specifically enlightening due to crucial differences or similarities on the lexical and the phrasal level. Arabic and Hebrew exhibit verb–object (VO) word order and therefore head–complement order associated with phrase final stress, cued by duration (Shlonsky, 1997). German has a mixed head–complement order with both, object–verb (OV) and VO, word orders being possible. This results in the occurrence of either finally or initially stressed phrases with duration cueing final and pitch/intensity cueing initial phrasal stress (Nespor et al., 2008). Therefore, if stress position within the phrase and its acoustic correlate is the relevant prosody level that affects perception, ITL-conforming grouping is expected for all three languages (as already evidenced for speakers of German). However, there are crucial differences in stress assignment on the lexical level across the three languages. Both German and Hebrew have lexical stress but with crucial differences. German shows a strong dominance of trochaic feet leading to a majority of initial stress in bisyllabic words (85% according to CELEX, Baayen et al., 1993) and penultimate stress in longer words. Hebrew, however, shows common word-final stress (iambic pattern), with iambic stress as the most predominant pattern in word tokens (72.2%) and types (75.5%), and a majority of final stress (58%) in longer words (Segal et al., 2009). The status of lexical stress is less clear in Arabic that has a weight-sensitive stress system with prominence typically falling on a syllable with a long vowel, which is often the first or penultimate syllable in a word (Jong & Zawaydeh, 1999). As a quantitative-sensitive stress system has also been proposed and is debated for German (c.f., Domahs et al., 2014) the stress system of Arabic may have more commonalities with the German than with the Hebrew one. Thus, if stress on the lexical level is crucial for the language-specific modulation of rhythm perception, differences are expected between the languages. Especially speakers of Hebrew, a language in which duration signals phrasal as well as lexical final stress, may show a general preference for a weak–strong grouping (Silver-Varod et al., 2016).

These hypotheses were tested with the (low-variability, no pauses) non-speech stimuli from Bhatara et al. (2016) with intensity and duration as the relevant acoustic cues. These cues were manipulated in four steps each to further investigate the impact of the acoustic salience on the responses (c.f. Wagner, 2022). As additional factors participants’ musical and second language experience were included as previous research has pointed to their influence (Boll-Avetisyan et al., 2016, 2017). The study was carried out as an online experiment. To control for potential effects of running the study online, German participants were also included to allow a method comparison with the German group from Bhatara et al. (2016) and, conversely, to test whether these results for German speakers could be replicated under the different testing conditions.

## Methods

### Participants

A total of 106 participants took part in the present study: Thirty-five native German speakers (recruited in Germany), and 36 native Arabic speakers and 35 native Hebrew speakers (both languages recruited in Israel). Age, demographic and background details are provided in Table 1.

**Table 1:** Participant characteristics by language group

		Arabic (n = 36)	German (n = 35)	Hebrew (n = 35)	Total (n = 106)
Gender	Female %(n)	50.0% (18)	71.4% (25)	71.4% (25)	64.2% (68)
	Male %(n)	50.0% (18)	25.7% (9)	28.6% (10)	34.9% (37)
	Other %(n)	0	2.9% (1)	0	0.9% (1)
Age (years)	M (SD)	22.8 (2.7)	25.8 (8.9)	24.2 (2.0)	24.3 (5.6)
	Min–Max	20–30	18–54	21–30	18–54
Education years^a^	M (SD)	15.6 (3.0)	12.7 (1.2)	12.2 (0.7)	13.5 (2.4)
	Min–Max	12–23	12–17	12–16	12–23
English as second language	Yes %(n)	91.7% (33)	100% (35)	91.4% (32)	94.3% (100)
Musical Experience^1 b^	M (SD)	1.72 (0.88)	2.17 (0.75)	1.42 (0.74)	1.77 (0.84)
	Min–Max	1–3	1–3	1–3	1–3

SD: standard deviation, n: number of participants, Min–Max: minimum–maximum, %: in percent

^a^Significant differences between the language groups in education years [F(2,103) = 33.393, *p* < .001]. In post hoc tests, the group of Arabic speakers had a significantly longer education compared to the groups of German and Hebrew speakers (p < .001), but no correlations were found between education and the dependent variable

^1^Musical experience was assessed on a 3-point scale, referring to low, medium and advanced experience

^b^Significant differences between language groups in musical experience [F(2,103) = 7.59, *p* < .001]. In post hoc tests, musical experience by German speakers was significantly higher compared to Hebrew and Arabic speakers (*p* < .001)

### Stimuli

The baseline “instrumental syllable” from the low-variability condition without pauses of Bhatara and colleagues (2016) was used to build all sequences for the present study, with an F_0_ of 200 Hz, an intensity of 60 dBA and a duration of 260 ms. The original authors termed this ‘chimera’ and created it by combining a trombone temporal structure with a cello spectrum (for details on the creation of the chimera see Agus et al., 2012 and Bhatara et al., 2016). To obtain different levels of prominence they manipulated this baseline chimera in four steps by either increasing intensity (+ 2, + 4, + 6 and + 8 dBA) or duration (+ 50, + 100, + 150, + 200 ms). The resulting instrumental syllables were used to form nine different sequences, each containing 32 instrumental syllables. The control sequence consisted of only the baseline chimera without any cues to prominence. The other eight sequences were created by combining the baseline chimera with one of the manipulated instrumental syllables, always alternating. For example, for the intensity condition with the lowest intensity difference the baseline chimera alternated with an instrumental syllable with 62 dBA. For a full description of the stimuli see Table 2.

**Table 2:** Overview of the acoustic stimuli used in the study

Condition	Step	Acoustic Cue Δ	Acoustic difference	Sequence duration [ms]	No. of sequences in test
Control	0	none		8320	40
Intensity	1	2 dB	60–62 dBA	8320	10
Intensity	2	4 dB	60–64 dBA	8320	10
Intensity	3	6 dB	60–66 dBA	8320	10
Intensity	4	8 dB	60–68 dBA	8320	10
Duration	1	50 ms	260–310 ms	9120	10
Duration	2	100 ms	260–360 ms	9920	10
Duration	3	150 ms	260–410 ms	10,720	10
Duration	4	200 ms	260–460 ms	11,520	10

The test stimuli (used for each participant) included a total of 120 sequences, 40 sequences in each condition (control, intensity, duration). Each step in the intensity and duration conditions was represented by 10 sequences. Half of the sequences in the intensity and duration conditions started with the strong syllables (those with increased intensity/duration), and the other half started with the weak syllables (the baseline chimera).

The sequences were masked over the first 3 s by white noise, which faded out with a raised-cosine function. At the same time, the sequence itself was faded in from silence, also with a raised-cosine function. We used this technique to avoid cues related to the first audible syllable in the sequence.

### Procedure

**Fig. 1:**
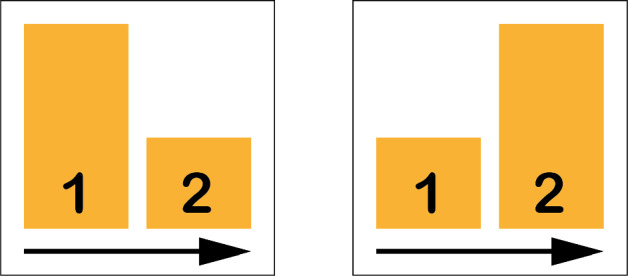
The two symbols used in the study to illustrate the rhythmic grouping: *Left panel*: The first sound is stronger/more prominent (i.e. strong–weak). *Right panel*: The second sound is stronger/more prominent (i.e. weak–strong). Because script direction in Arabic and Hebrew is right to left, we included an arrow to make the direction transparent

Participants performed the study at home using their personal computer due to COVID-19 restrictions. They were instructed to perform the test in a quiet room, to use headphones, and to choose the most comfortable volume level for listening on their computer. Each participant received a link to the LabVanced platform (Finger et al., 2017) where the study was implemented. First, the participants completed a short questionnaire that included questions on age, language, education, hearing, language disabilities and musical experience. Next, the auditory task was presented with the following description: “When people hear a sequence of two sounds, one of the sounds tends to be perceived as more strong/dominant/prominent. In this study we are interested in your perception of sound sequences. There is no right or wrong answer—it is all about your impression. We will use two symbols to illustrate this perception”. Figure 1 shows the buttons for the two perception choices. The side of the symbols was counterbalanced across participants.

Then participants were presented with a sequence of two pure tones (a beep sound), which were similar to the test stimuli in duration and intensity (base tone: 260 ms, 60 dBA, 334 Hz). Contrary to the experimental stimuli, the prominent tone in this instruction example was marked by a combination of three all cues: duration (+ 200 ms), intensity (+ 8dBA) and pitch (+ 17 Hz). The first two-tone sequence was a trochee (prominence on the first tone), the second an iamb (prominence on the second tone). After the explanation of the response symbols using the two sound examples there was a practice part, in which these pure tones were presented as longer sequences (like the experimental stimuli) comprising of 32 pure tones with masking noise at the beginning in a practice block. For this practice block four sequences were used, each presented twice in randomised order resulting in a total of eight sequences being presented. In two sequences prominence was marked by all three cues (intensity, pitch and duration), in the other two by pitch only. Half of the sequences started with a stressed tone, the other half with an unstressed tone (just like in the experimental sequences; note that the beginning was masked). Participants were instructed to listen carefully to each sequence of sounds. They were told that each sequence consisted of sounds that alternated in salience, creating a two-beat rhythm. Their task was to report whether, in these groups of two, the salient sound was in the first or second position. By pressing the left or right arrow key on the keyboard (corresponding to the side of the perception symbol, see Fig. 1) as quickly as possible (while the sound sequence was still playing). Instructions were written in the native language and were equivalent in Arabic, German and Hebrew. No feedback was given.

After the practice block the participants were presented with the 120 experimental sequences. After 40 trials, participants were allowed to take a small break if they wished. The stimuli were presented in randomised order. The procedure was approved by the ethics committee of TAU.

## Analysis and Results

### Data Analysis

A generalized linear mixed logit model was performed using the statistics program SPSS. The dependent variable was ITL conformity according to the predicted response, that is, whether the response followed the ITL or not (i.e., strong–weak [trochaic] = 1 and weak-strong [iambic] = 0 for intensity-varied and control sequences; trochaic = 0 and iambic = 1 for duration-varied sequences). We chose strong–weak intensity-like grouping as the expected pattern for the control sequences because of previous work suggesting that trochaic grouping might be the default in invariant sequences (Bhatara et al., 2013; Bolton, 1894; Hayes, 1995). The factors in the analysis included the between-subject variable native language (Arabic, German or Hebrew), and the within-subject variables were condition (duration, intensity or control), step (4 steps), the side of strong–weak and weak–strong response (Response side) and the prominence of the first syllable, i.e., whether sequences started with a strong or a weak syllable (First syllable) as well as all interactions among these factors. A random intercept for participants was included. In addition, musical experience was also included as a fixed factor based on previous studies (e.g., Bhatara et al., 2016). For condition, step and language Bonferroni pair-wise contrasts were used to examine the differences between the levels.

### Results

#### Responses against ITL predictions

In order to answer the question whether each group of participants responded according to the ITL, we first compared the number of expected ITL-conforming responses with chance level. A series of one-sample *t*-tests for each language and condition showed that for German speakers, responses for both the duration (*M* = 0.69) [*t* (34) = 5.57, *p* < 0.001] and the intensity condition (*M* = 0.63) [*t* (34) = 3.59, *p* < 0.001] were significantly above chance. For Hebrew speakers, ITL-conforming responses for both control (*M* = 0.64) [*t* (34) = 4.89, *p* < 0.001] and duration (*M* = 0.73) [*t* (34) = 9.75, *p* < 0.001] were significantly above chance. For Arabic speakers, however, only ITL-conforming responses for the duration condition (*M* = 0.64) were significantly above chance [*t* (35) = 3.58, *p* < 0.001]. Overall, the results suggest that changes in duration were judged according to the ITL prediction across the three languages, whereas changes in intensity were judged according to the ITL prediction only by German speakers.

#### Group comparison

In order to compare the groups directly, a mixed model was fitted. Full results of the mixed model are shown in Supplement 1. Fixed effects are described in Table 3.

**Table 3:** Fixed effects with correct response as the dependent variable

Effect	F	*p*
Musical experience	F(1,12,558) = 2.755	.097
Response side	F(1,12,558) = 0.002	.963
Condition	F(2,12,558) = 77.923	< .001
Step	F(3,12,558) = 8.447	< .001
First syllable of the sequence	F(1,12,558) = 0.493	.482
Condition * step	F(6,12,558) = 19.114	< .001
Condition * first	F(2,12,558) = 3.886	.021
Language	F(2,12,558) = 0.886	.412
Language * condition	F(4,12,558) = 12.827	< .001
Language * step	F(6,12,558) = 0.760	.601
Language * condition * step	F(12,12,558) = 1.150	.314

**Fig. 2:**
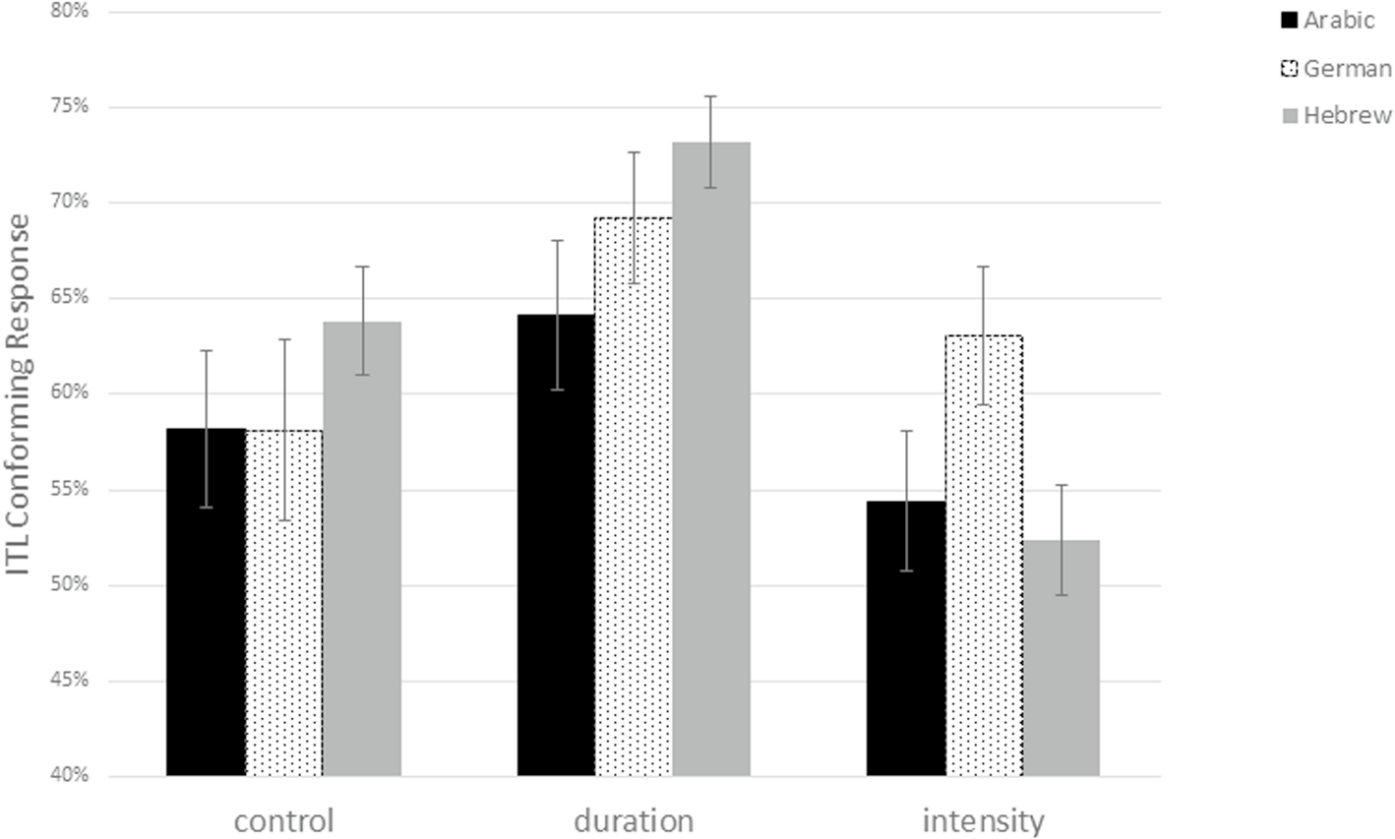
Estimated correct responses in the three conditions by all three language groups resulting from the model

As shown in Table 3, a main effect was found for condition (*p* < 0.001). Bonferroni pairwise contrasts showed significant differences between ITL-conforming responses in the duration condition (*M* = 0.71) and the control condition (*M* = 0.62; *p* < 0.001), between ITL-conforming responses in the duration condition and the intensity condition (*M* = 0.58; *p* < 0.001), and between the intensity and the control conditions (*p* < 0.001). There were no effects of Language, Response side, First syllable of the sequence and musical experience. An interaction was found between Language X Condition (*p* < 0.001; Fig. 2).

Bonferroni pair-wise contrasts demonstrated significant differences only in the duration condition between speakers of Arabic (*M* = 0.66) and Hebrew (*M* = 0.76; *p* = 0.01). Borderline significance was found in the intensity condition between speakers of Hebrew (*M* = 0.54) and German (*M* = 0.64; *p* = 0.05). No significant Language X Step interaction, or Language X Condition X Step interaction were found.

**Fig. 3:**
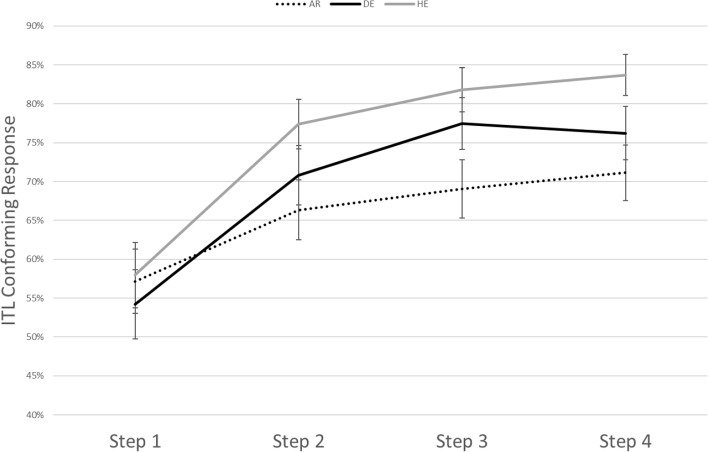
Estimated correct responses split by step and language group in the duration condition resulting from the model

**Fig. 4:**
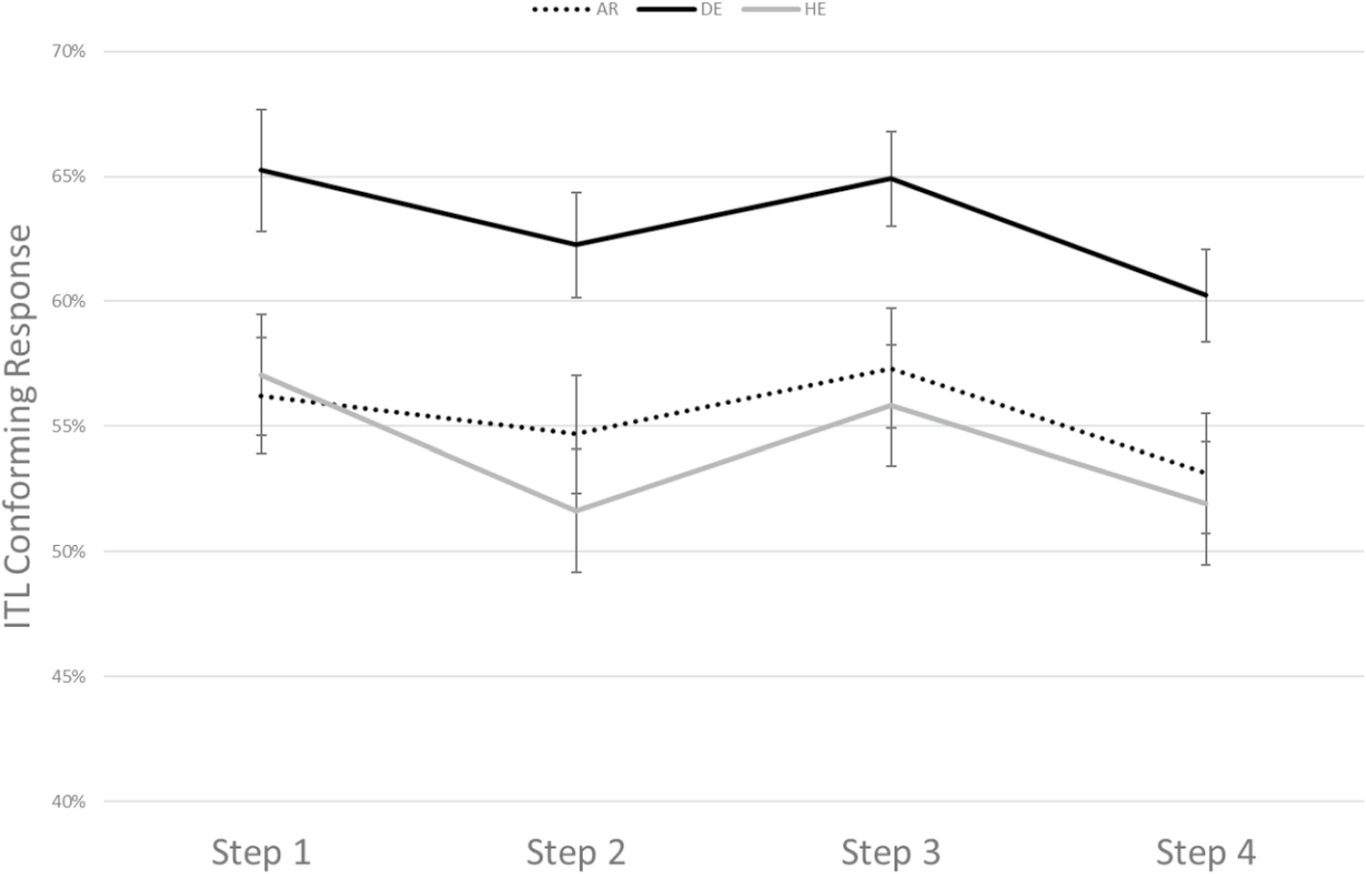
Estimated correct responses split by step and language group in the intensity condition resulting from the model

There was also a main effect for step (*p* < 0.001), with significant differences between ITL-conforming responses in step 1 (*M* = 0.60) and step 2 (*M* = 0.64), and between step 1, step 3 (*M* = 0.66) and step 4 (*M* = 0.65; *p* < 0.01). No significant differences were found between the other steps. An interaction was found between Condition X Step (*p* < 0.001). Significant differences between steps could occur only in the duration and the intensity conditions as shown in Figs. 3 and 4.

Bonferroni pair-wise contrasts for the duration condition demonstrated significant differences between the ITL-conforming responses in step 1 (*M* = 0.56) and 2 (*M* = 0.72; *p* < 0.001), between step 1 and 3 (*M* = 0.77; *p* < 0.001), between step 1 and 4 (*M* = 0.78; *p* < 0.001), between step 2 and 4 (*p* = 0.002), and between step 2 and 3 (*p* = 0.01). In the intensity condition a significant difference was only found between step 1 (*M* = 0.60) and step 4 (*M* = 0.55; *p* = 0.04).

A further interaction was found between Condition X First syllable (*p* = 0.02) with significantly more ITL-conforming responses in the duration condition, when the sequence started with a long syllable (*M* = 0.73) compared to the short syllable (*M* = 0.69; *p* = 0.01).

## Discussion

Our study set out to further investigate whether cross-linguistic differences in rhythm perception that have been found in previous research are mainly related to differences between languages in their instantiation of stress at the lexical level as suggested by Bhatara et al., (2013, 2016) or at the phrasal level as suggested by Langus et al. (2016). To contribute to this discussion we compared grouping of non-speech stimuli by speakers of three different languages that have similar prosodic patterns on the level of the phonological phrase but are different with respect to their lexical stress systems.

Interestingly, our results show ITL-conforming groupings in the duration condition across the three languages under investigation but larger cross-linguistic differences in the intensity and the control condition. This finding is not in line with previous research that found crosslinguistic differences in the perception of duration but not for intensity. The following discussion will focus on the question whether our findings pattern with differences across the languages that we tested and how they relate to previous research.

### Intensity

Concerning intensity, only German speakers showed above-chance performance in judging intensity-changing sequences as strong–weak. However, comparing across languages, this trochaic bias in native German speakers tended to be stronger than in Hebrew speakers but was not statistically different from the Arabic speakers. Furthermore, no difference between Hebrew and Arabic speakers was obtained. The result for the German speakers is clearly in line with the ITL which predicts strong–weak grouping if intensity is the cue for acoustic prominence and congruent with previous studies with German speaking participants (Bhatara et al., 2013, 2016). It is also in line with other studies on trochaic languages including English and Spanish (e.g., Crowhurst, 2016). The fact that speakers of Hebrew do not show this strong–weak bias in this condition could be explained by the fact that on both levels—lexical as well as phrasal—Hebrew units show final prominence with duration as the relevant acoustic cue (Silver-Varod et al., 2016). However, the Hebrew speakers in the study did not treat every salient syllable—independently of its acoustic properties—as a final syllable of a unit, as in this case they should show a lower than chance performance in the intensity condition by grouping the sequences into weak–strong groups. Thus, it is possible that Hebrew speakers demonstrate a combination of different tendencies: a general acoustic bias to judge intensity-changing sequences as strong–weak and a language specific bias to judge salient-changing sequences as weak–strong. The pattern of the Arabic speakers lies somehow in between that of the German and Hebrew speakers – like the Hebrew speakers, they do not show above-chance performance in the intensity condition, but they also do not show a lower number of ITL-conforming responses in this condition than the German speakers. As said above, Arabic has a weight-sensitive system such that syllables with a long vowel attract stress which usually is the penultimate syllable (Jong & Zawaydeh, 1999). This system resembles a trochaic pattern but with duration as a relevant cue which may—similarly to what has been observed for speakers of Zapotec (Crowhurst & Teodocio Olivares, 2014)—work against ITL-conforming grouping in the intensity condition.

What is interesting about the results in the intensity condition is that the magnitude of the acoustic difference between the syllables (i.e., the step) does not have a large impact on the participants’ performance and that this is independent of the language background of the participant. The only effect of step concerned the steps with the largest difference in intensity (step 1 vs. step 4) while there were no statistical differences in the responses to any of the other steps. Interestingly, this effect goes into a negative direction, that is, the smaller increase of intensity in step 1 was associated to a larger amount of strong–weak responses than the larger increase of intensity in step 4. This resembles findings by Crowhurst (2016) who found the largest increase of strong–weak groupings by English and Spanish speaking listeners when the acoustic difference between the syllables was rather small compared to a control condition without intensity differences. As there are no interactions with language concerning the step effect in our data we may conclude that there are no principal differences between the language groups regarding their perception of the relevant acoustic property. This could suggest that pure acoustic sensitivity towards the acoustic cue is not the crucial factor that forms the basis of the cross-linguistic differences in our results but that these occur at the phonological level of processing the stimuli.

### Duration

The most consistent finding across the three language groups relates to the duration sequences as these were judged as weak–strong more often than predicted by chance, independent of the language background. However, differences across languages occurred in the strength of this bias, as native Hebrew speakers judged sequences changing in duration more frequently as iambic than did native Arabic speakers. The performance of the German speakers was between the two other language groups but did not differ significantly from either. The strong preference that Hebrew speakers show in the duration condition can be related to the fact that the Hebrew language consistently marks word and phrasal stress by a long duration on the last element of the unit. Thus, duration as an indication for a weak–strong grouping is supported by prosodic properties on the lexical as well as on the phrase level in Hebrew. This “double” support is not present in German and Arabic as these both do not show this parallel in the stress systems of the lexical and the phrasal level (as Hebrew does).

Although statistically not different from German, speakers of Arabic show a comparably weak ITL-conforming response to the duration cue. Again, this could be due to the fact that stressed syllables are typically long but non-final. Additionally, as vowel duration is phonemic in Arabic, the concept of lexical stress, which distinguishes between two similar words, may not be intuitive for Arabic speakers. Interestingly, in the study with identical material, the French participants—as speakers of a language without lexical stress—showed fewer ITL-conforming responses overall than the German participants (Bhatara et al., 2016), which is similar to the pattern of the Arabic speakers in the present study. Again, this observation is compatible with the assumption that properties of the lexical stress system modulate ITL effects on grouping.

The magnitude of the duration cue manipulation had some fine-grained effects. A duration difference of 50 ms led to a lower proportion of weak–strong responses than a difference of 100, 150 or 200 ms. In addition, a difference of 100 ms led to a lower proportion of weak–strong responses than a difference of 150 or 200 ms. As there was no interaction between step and language, our data do not provide any indication that the language groups were differently sensitive to the duration cue in their acoustic processing. Thus, we may conclude that the large weak–strong bias that the Hebrew speakers show in the duration condition is not related to a larger acoustic sensitivity to durational differences but instead to the status of the cue in the phonological system of the Hebrew language and therefore arises on a higher (phonological) level of processing.

### Control

The control condition was included in the experiment as it—given that there are no prominence differences between syllables in the string—was thought to provide the strongest evidence for the impact of phonological knowledge on rhythmic grouping. This assumption was mainly based on previous findings showing that speakers of German showed a trochaic bias in the control condition while speakers of French did not (Bhatara et al., 2013). Based on these findings we expected Hebrew speakers to show a weak–strong bias in the control condition because this would correspond to the dominant pattern in their language.

However, the results from the control condition did not match our expectations in two aspects. First, Hebrew speakers did not show a weak–strong bias but, contrary to our assumptions, a strong–weak bias in the control condition. As far as we are aware, the only study beyond the above-mentioned study with German participants that included a control condition is the study by Hay and Diehl (2007) that tested English and French listeners. Interestingly, they found a trochaic bias in the control condition for both language groups with speech as well as non-speech stimuli. However, this bias was modulated by context: Hay and Diehl presented their intensity and duration stimuli in blocks with control sequences included in the blocks. The trochaic bias only occurred within the duration block but was not observed in the intensity block. This finding as well as our result from Hebrew speakers thus might support the proposal by Hayes (1995) according to which sequences that do not vary in duration are perceived as strong–weak. This tendency may be particularly strong if duration is specifically salient in the experiment (due to the blocking in the Hay and Diehl study) or in the language of the listener (as for the Hebrew speakers in the current study).

However, the question remains why the German listeners did not show a significant trochaic bias in the control condition as they did before in other experiments. Besides the fact that this was the first online study on the ITL, there was one major difference between the present study and the previous studies with German participants, specifically with the study by Bhatara et al. (2016) that used the same materials. In that study the control sequences were presented much less frequently than the duration and intensity sequences (10/40/40). In our study the ratio of control, duration and intensity sequences was equal, with 40 sequences in each condition. This high number of control sequences may have made the participants aware that there are sequences without any acoustic differences between the syllables and that this affected their responses or they may have avoided to the select the strong–weak response in the overwhelming number of cases.

### Relation of Findings to Previous Research

In contrast with previous studies, cross-linguistic differences in this study were mostly pronounced in the intensity condition, with only the German participants showing ITL-conforming grouping there while all groups showed ITL-conforming grouping in the duration condition. In contrast, results from previous studies have shown stronger cross-linguistic variation in the duration condition compared to the intensity condition. Langus et al. (2016) found ITL-conforming weak–strong grouping in Italian, but ITL-non-conforming strong–weak groupings with speakers of Turkish and Persian for linguistic sequences varying in duration. For pitch—the second cue considered in their study—all language groups grouped ITL conform strong–weak. However, no cross-linguistic differences but ITL-conforming grouping in the duration as well as the pitch condition were found with non-speech stimuli. Similarly, Iversen et al. (2008) report cross-linguistic differences between English and Japanese speakers only in the duration condition, in which English speakers showed ITL-conforming grouping but no consistent grouping in Japanese speakers. What is interesting is, that in these two studies all languages that show ITL-non-conforming patterns in duration (Turkish, Persian, Japanese) are languages with head–complement order and therefore phrase-initial stress – that typically is not signalled by duration but by pitch and intensity. In contrast, all languages tested so far with complement–head order and therefore phrase-final stress with typically duration as the acoustic cue show ITL-conforming grouping in the duration condition. The two new languages tested in the current study (Arabic and Hebrew) perfectly fit into this picture as both have head–complement order with phrasal-final stress and their speakers show ITL-conforming grouping in the duration condition. Coming back to our question about which level generates cross-linguistic effects, this observation may favour the proposal that stress on the phrasal level is the central factor for generating cross-linguistic differences in rhythm perception.

What does not fit to any of the previous studies is the ITL-non-conforming results of the Arabic and Hebrew speakers for the intensity-varying sequences, with chance-level performance for both language groups in this condition. We cannot exclude that the online implementation of the task has contributed to this result as we could not control for loudness in the home equipment. The perception of the relatively small dB differences in the intensity condition may have been specifically affected by this methodological drawback and as a result contributed to only chance-level performance in the Arabic and Hebrew speakers. One result supporting this reasoning is that the German speakers in the current experiment showed overall smaller differences between the conditions (duration, intensity and control) than in the original Bhatara et al. (2016) study.

Overall, our study contributes to observations that rhythmic grouping may be affected by properties of the native language of the listeners by extending the findings to two new languages. Most importantly, our findings indicate that the question which level of the prosodic hierarchy is relevant for generating these language-specific effects may be the wrong one. In contrast, they support the assumption that prominence pattern at the level of the phonological phrase (which are related to syntactic word order properties of a language) as well as at the level of the prosodic foot (being mostly relevant for stress patterns on the word level) both can have an impact on ITL-like grouping. Integrating our results into the findings of previous research, we would tentatively suggest that prominence pattern on the level of the phonological phrase mostly modulate the effects of duration on grouping as ITL-non-conforming grouping was only found in languages with phrase-initial stress (for Japanese,: Iversen et al., 2008; for Persian and Turkish; Langus et al., 2016). However, cross-linguistic differences were also found for languages that show the same prominence pattern in the phonological phrase but differ in their lexical stress systems. Properties that seem to be relevant in this respect are for example the presence of lexical stress (French: Bhatara et al., 2013, 2016; maybe also Arabic), the position of the main word stress position (Hebrew in our study) or the acoustic cues that mainly mark stress (Zapotec: Crowhurst & Teodocio Olivares, 2014; Arabic in our study). Future research should focus on languages that have phonological properties that are particularly informative concerning the specific impact that features of these different levels of the prosodic hierarchy have on rhythmic grouping.

### Implications

The main contribution of the current study lies in extending the knowledge on the iambic-trochaic law to Arabic and Hebrew, two languages, that to the best of our knowledge have not been studied in this regard. The findings highlight the language-specific influence of the native language on grouping strategies in adults. These strategies may have important implications for second language learners who need to learn the prosodic characteristics of the new language in order to segment and produce linguistic units. Incorporating examples of clear and even exaggerated prosodic patterns of words, sentences and phrases in the second language may assist novice learners to identify words and phonological phrases in running speech and overcome the influence of the first language on rhythmic grouping of linguistic units. In order to further expand our knowledge on the influence of the native language on the ITL, more cross-linguistic studies on infants are recommended. Language-specific changes in how the ITL applies throughout development will have consequences on early segmentation of words from fluent speech and are therefore important for language acquisition in mono- and multilingual infants.

## Supplementary Information

Below is the link to the electronic supplementary material.

Supplementary file1 (DOCX 14 kb)
