# Supplementary material for: Rhythm Perception in Speakers of Arabic, German and Hebrew
Source: J Psycholinguist Res. 2025 Jan 5;54(1):5. doi: 10.1007/s10936-024-10121-5 (PMC11700906; doi:10.1007/s10936-024-10121-5)
Supplement: Supplementary file 1 — Supplementary file1 (DOCX 14 kb) [file 10936_2024_10121_MOESM1_ESM.docx]

**Supplement 1**

Results of overall analysis

| Correct Response | β  (Estimate) | Standard Error | t | p |
| --- | --- | --- | --- | --- |
|  |  |  |  |  |
| Intercept | -.252 | .2423 | -1.040 | .298 |
| Right vs. Left (Response side) | .016 | .1562 | .101 | .919 |
| Duration vs. Control (D-C) | .579 | .1650 | 3.505 | **.000** |
| Intensity vs. Duration (I-D) | 1.694 | .1857 | 9.125 | **.000** |
| Step 1 vs. Step 2 (Step 1-2) | .207 | .1561 | 1.325 | .185 |
| Step 2 vs. Step 3 (Step 2-3) | -.012 | .1558 | -.078 | .938 |
| Step 3 vs. Step 4 (Step 3-4) | .158 | .1560 | 1.013 | .311 |
| Strong vs. weak (First syllable) | .088 | .0661 | 1.338 | .181 |
| Arabic vs. German (Language A-G) | .052 | .2422 | .213 | .831 |
| German vs. Hebrew (Language G-H) | .340 | .2587 | 1.313 | .189 |
| Musical experience (Music) | .155 | .0998 | 1.552 | .121 |
| D-C*Step 1-2 | -.076 | .2249 | -.338 | .735 |
| D-C*Step 2-3 | .103 | .2244 | .460 | .645 |
| D-C*Step 3-4 | -.209 | .2235 | -.936 | .349 |
| I-D*Step 1-2 | -1.521 | .2377 | -6.401 | **.000** |
| I-D*Step 2-3 | -.393 | .2448 | -1.605 | .109 |
| I-D*Step 3-4 | -.291 | .2497 | -1.165 | .244 |
| D-C* First syllable | -.068 | .0939 | -.728 | .466 |
| I-D* First syllable | -.272 | .0973 | -2.794 | **.005** |
| Language A-G*D-C | -.215 | .2246 | -.959 | .338 |
| Language G-H*D-C | -.828 | .2313 | -3.581 | **.000** |
| Language A-G*I-D | -.782 | .2426 | -3.221 | **.001** |
| Language G-H*I-D | -.745 | .2545 | -2.928 | **.003** |
| Language A-G*Step 1-2 | -.081 | .2224 | -.366 | .714 |
| Language G-H*Step 1-2 | .029 | .2322 | .125 | .900 |
| Language A-G*Step 2-3 | .075 | .2221 | .336 | .737 |
| Language G-H*Step 2-3 | .173 | .2315 | .748 | .454 |
| Language A-G*Step 3-4 | .012 | .2225 | .053 | .958 |
| Language G-H*Step 3-4 | .048 | .2319 | .206 | .837 |
| Language A-G*D-C*Step 1-2 | -.088 | .3184 | -.276 | .782 |
| Language G-H*D-C*Step 1-2 | .240 | .3299 | .728 | .466 |
| Language A-G*D-C*Step 2-3 | -.256 | .3178 | -.804 | .421 |
| Language G-H*D-C*Step 2-3 | .019 | .3288 | .059 | .953 |
| Language A-G*D-C*Step 3-4 | -.114 | .3173 | -.358 | .720 |
| Language G-H*D-C*Step 3-4 | .258 | .3283 | .787 | .431 |
| Language A-G*I-D*Step 1-2 | .783 | .3296 | 2.375 | **.018** |
| Language G-H*I-D*Step 1-2 | .202 | .3443 | .588 | .556 |
| Language A-G*I-D*Step 2-3 | .108 | .3360 | .320 | .749 |
| Language G-H*I-D*Step 2-3 | -.094 | .3518 | -.268 | .789 |
| Language A-G*I-D*Step 3-4 | .022 | .3404 | .065 | .948 |
| Language G-H*I-D*Step 3-4 | .159 | .3591 | .444 | .657 |
